# Disinhibition of the HECT E3 ubiquitin ligase WWP2 by polymerized Dishevelled

**DOI:** 10.1098/rsob.150185

**Published:** 2015-12-23

**Authors:** Thomas Mund, Michael Graeb, Juliusz Mieszczanek, Melissa Gammons, Hugh R. B. Pelham, Mariann Bienz

**Affiliations:** MRC Laboratory of Molecular Biology, Cambridge Biomedical Campus, Francis Crick Avenue, Cambridge CB2 0QH, UK

**Keywords:** Dishevelled polymerization, NEDD4 family HECT E3 ubiquitin ligases, DEP domain, Notch, Wnt

## Abstract

Dishevelled is a pivot in Wnt signal transduction, controlling both β-catenin-dependent transcription to specify proliferative cell fates, and cell polarity and other non-nuclear events in post-mitotic cells. In response to Wnt signals, or when present at high levels, Dishevelled forms signalosomes by dynamic polymerization. Its levels are controlled by ubiquitylation, mediated by various ubiquitin ligases, including NEDD4 family members that bind to a conserved PPxY motif in Dishevelled (mammalian Dvl1–3). Here, we show that Dvl2 binds to the ubiquitin ligase WWP2 and unlocks its ligase activity from autoinhibition. This disinhibition of WWP2 depends on several features of Dvl2 including its PPxY motif and to a lesser extent its DEP domain, but crucially on the ability of Dvl2 to polymerize, indicating that WWP2 is activated in Wnt signalosomes. We show that Notch intracellular domains are substrates for Dvl-activated WWP2 and their transcriptional activity is consequently reduced, providing a molecular mechanism for cross-talk between Wnt and Notch signalling. These regulatory interactions are conserved in *Drosophila* whose WWP2 orthologue, Suppressor-of-deltex, downregulates Notch signalling upon activation by Dishevelled in developing wing tissue. Attentuation of Notch signalling by Dishevelled signalosomes could be important during the transition of cells from the proliferative to the post-mitotic state.

## 1. Introduction

Wnt/β-catenin signalling controls normal animal development and tissue homeostasis, and aberrant activation of the pathway leads to many cancers, most notably colorectal cancer [1]. The OFF state of this signalling pathway is determined largely by modification of its key effector β-catenin with ‘canonical’ lysine 48-linked ubiquitin (K48-Ub) [2]: in the absence of Wnt stimulation, β-catenin is continuously targeted for proteasomal degradation by an SCF E3 ubiquitin ligase, whose β-TrCP adaptor recognizes a phosphodegron in its N-terminus. This phosphorylation mark is imparted by the serine/threonine kinases of the Axin degradasome (i.e. glycogen synthase kinase 3 and casein kinase 1*α*) whose assembly and function depends on the APC tumour suppressor [3–5]. Upon binding of Wnt ligand to its Frizzled receptor and LRP5/6 co-receptor, the Axin degradasome is recruited into a signalosome assembled by Dishevelled (Dvl, or Dsh in *Drosophila*) at the cytoplasmic face of Frizzled and LRP5/6, which blocks its activity. Thus, β-catenin is stabilized and accumulates, to co-activate a Wnt-specific transcriptional programme in the nucleus [6,7]. However, Dishevelled can also signal through non-nuclear (β-catenin-independent) Wnt signalling branches, to control cytoplasmic events such as planar cell polarity (PCP) and cell movement [8]. Nuclear and non-nuclear Wnt signalling branches are mutually exclusive, owing to Dishevelled which acts as a pivot between them.

To assemble a signalosome, Dishevelled undergoes dynamic head-to-tail polymerization through its DIX domain which can be triggered by Wnt binding to its receptors, or by high expression levels [7,9]. To avoid inadvertent signalling, its levels must therefore be kept low, which is achieved by ubiquitylation by various E3 ligases, including a Cullin 3 ubiquitin ligase complex which targets it for proteasomal degradation [10]. Similarly, Dvl can be targeted for proteasomal degradation by the anaphase-promoting complex (APC/C) which recognizes a D-box in its DEP domain [11] and, notably, by several members of the NEDD4 family of HECT ubiquitin E3 ligases (including Itch, NEDD4L, NEDD4 and Smurf2) which bind to a highly conserved PPxY motif in Dvl via their WW domains [12–16]. Itch- and Smurf2-dependent ubiquitylation of Dvl correlates with its phosphorylation [12,13], which is acquired during signalling [17–19], suggesting that Dvl is downregulated by these ligases during or after signalling. Indeed, some of these ligases seem to specifically target the PCP signalling function of Dvl [11,12], underscoring the notion that the levels of Dvl need to be tightly regulated in post-mitotic cells.

In addition to targeting Dvl for proteasomal degradation, ubiquitylation of its DIX domain, for example by the HECT E3 ligase HUWE1 [20], blocks its polymerization [21] and thus attenuates Dvl's signalling function without destabilizing it. The ubiquitylation imparted on Dvl by HUWE1 involves predominantly K11-Ub and K63-Ub linkages [20], similarly to ubiquitylation by the NEDD4 family ligases which also generate these ‘non-canonical’ ubiquitin linkages [22]. Consistent with this, the de-ubiquitinase (DUB) USP14 appears to stimulate the signalling activity of Dvl by trimming its K63-linked ubiquitin chains [23]. By contrast, another K63-specific DUB called CYLD attenuates its signalling activity [24], which implies that ubiquitylation can also have a positive impact on Dvl.

Examining the ubiquitylation of Dvl2 by mass spectroscopy, we found K11-Ub in the absence of Wnt, and additionally K63-Ub and K48-Ub after Wnt stimulation, consistent with previous reports [20,24]. Because each of these ubiquitin linkages can be generated by NEDD4 family E3 ligases [22], they could be the enzymes imparting these ubiquitylations. Here, we test the interaction between Dvl2 and members of the human NEDD4 family, and identify WWP2 as a preferred binding partner. Like its relatives, WWP2 binds to the conserved PPxY motif of Dvl2, but also interacts with the DEP domain, thereby ubiquitylating and destabilizing Dvl2. Modification by WWP2 requires activation of the ligase by Dvl2; like other NEDD4 family members, WWP2 is autoinhibited, and this is relieved by Dvl2 in an analogous manner to the activation of NEDD4 family ligases by Ndfips (NEDD4 family-interacting proteins) [25]. We refer to this reversal of autoinhibition as disinhibition. Notably, disinhibition of WWP2 by Dvl2 depends on its polymerization, and is thus contingent on signalosome formation. The same is true for the *Drosophila* WWP2 homologue Suppressor-of-deltex (Su(dx)), whose disinhibition also requires polymerization-competent Dsh. Both Dsh and Su(dx) have been implicated in the regulation of Notch signalling, and our evidence suggests that disinhibition of Su(dx) by Dsh attenuates Notch signalling in developing *Drosophila* wings. We further show that the intracellular domain (NICD) of different mammalian Notch isoforms can be ubiquitylated by Dvl2-activated WWP2. We propose that the disinhibition of WWP2/Su(dx) by Dishevelled signalosomes serves to downregulate Notch signalling.

## 2. Results

### 2.1. Dvl2 interacts preferentially with WWP2

**Figure 1.**
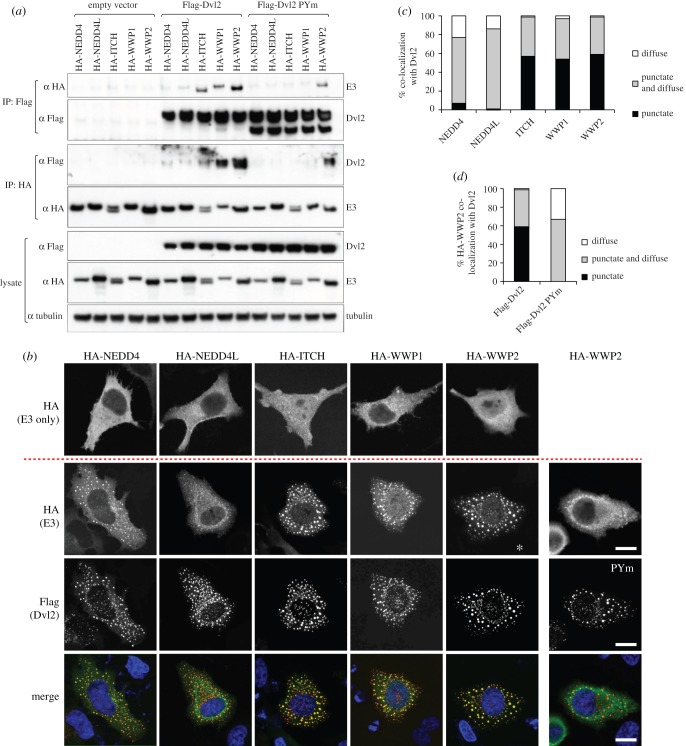
Preferred association between Dvl2 and WWP2. (*a*) Western blots of coIPs from HEK293ET cells co-transfected with wt or PPxY mutant Flag-Dvl2 and different members of the NEDD4 family (tagged with haemagglutinin (HA)), showing PPxY-dependent association of Dvl2 with these E3 ligases except for WWP2 whose high affinity for Dvl2 only partially depends on its PPxY element. (*b*) Single confocal sections of representative HeLa cells co-transfected with Dvl2 and the indicated E3 ligases (bottom three rows) or the ligases alone (upper row), and co-stained with α Flag (red) and α HA (green), showing efficient recruitment of cytoplasmic WWP2 into the punctate Dvl2 signalosomes (as judged by the almost complete absence of diffuse HA staining, asterisk), which is blocked by the PPxY mutation (PYm); blue, DAPI stain; scale bars, 10 µM. (*c*,*d*) Quantification of IF data shown in (*b*); 100 cells were scored in each case and assigned to one of three classes (punctate, e.g. WWP2; punctate and diffuse, e.g. NEDD4; diffuse, e.g. NEDD4L).

To test interactions between Dvl2 and different NEDD4 family members we used co-immunoprecipitation (coIP) assays in HEK293ET cells co-transfected with Flag-Dvl2 and individual HA-tagged NEDD4 ligases (figure 1*a*). This revealed that Dvl2 robustly coIPs with WWP2, and vice versa, and also with the most closely related ligases WWP1 (more than 80% identical to WWP2) and Itch, but barely at all with NEDD4 and NEDD4L. These interactions were largely dependent on the PPxY motif of Dvl2, but in the case of WWP2 some binding could still be observed even when PPxY was mutated (Dvl2-PYm), implying additional interactions beyond the canonical PPxY–WW binding (figure 1*a*). This suggests that WWP2 has evolved as a favoured partner of Dvl2.

Interactions could also be demonstrated by immunofluorescence: NEDD4 family ligases are distributed diffusely throughout the cytoplasm, whereas overexpressed Dvl2 forms distinct cytoplasmic puncta (corresponding to Wnt-independent signalosomes) [9], which recruit axin to block its activity in promoting β-catenin degradation [26]. When co-expressed with Dvl2, the ligases WWP2, WWP1 and Itch were relocated efficiently into the Dvl2 puncta, whereas NEDD4 and NEDD4L remained mostly diffuse (figure 1*b*). Dvl2-PYm was also punctate, but failed to recruit WWP2 efficiently into the puncta, indicating that the PPxY interaction is a major contributor to binding. Quantification of these assays confirmed the preference of Dvl2 for the WWP2/1/Itch subgroup versus other NEDD4 family members, and underscored the importance of the PPxY motif of Dvl2 for its interaction with these E3 ligases (figure 1*c,d*).

### 2.2. Dvl2 disinhibits WWP2

**Figure 2.**
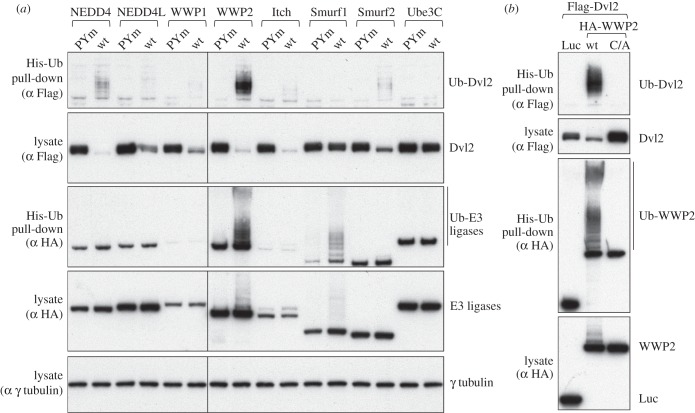
Disinhibition of WWP2 ligase by Dvl2. Western blots of His pull-downs from HEK293T cells co-transfected with His-Ub, Flag-Dvl2 (wt or PPxY mutant) and HA-tagged wt or catalytically dead (C/A) NEDD4 family ligases, as indicated above panels. WWP2 autoubiquityation is induced by wt but not PPxY-mutant Dvl2 (*a*); note that the levels of ubiquitylation of Dvl2 do not strictly correlate with its destabilization, and that autoubiquitylated WWP2 appears to be completely stable (see also figure 6). Dvl2-dependent autoubiquitylation of WWP2 depends on its catalytic activity (*b*). Luciferase (Luc) was expressed as a negative control for WWP2.

To assess the ubiquitylation of Dvl2 by various ligases, we co-expressed them with His-ubiquitin (His-Ub), allowing us to monitor specifically the ubiquitylated Dvl2 after affinity purification by nickel resin under denaturing conditions (‘His pull-down’). We also tested the PPxY-mutated version of Dvl2. With the exception of Smurf1, all the NEDD4 family ligases significantly reduced the level of Dvl2 in a PPxY-dependent manner, as expected from previous work. However, ubiquitylation of Dvl2 was particularly striking with WWP2 (figure 2*a*). In contrast, expression of Ube3C, a HECT ligase that lacks WW domains, resulted in neither ubiquitylation nor degradation of Dvl2.

Interestingly, we observed a striking induction of WWP2 autoubiquitylation on co-expression with Dvl2, and this was also largely dependent on the PPxY motif (figure 2*a*). This ubiquitylation is catalysed by WWP2 itself, because it is abolished by a C/A mutation at the active site (figure 2*b*). Such PPxY-dependent disinhibition of autoubiquitylation is similar to that induced by the Ndfip proteins, but whereas the Ndfips are active on many NEDD4 family ligases [25], the Dvl2 effect was only readily apparent with WWP2, and to a lesser extent with Smurf1. The prominent effect of Dvl2 on WWP2, together with its ability to associate with WWP2 even when its PPxY motif was mutated, prompted us to examine the interactions between Dvl2 and WWP2 in more detail.

### 2.3. Disinhibition of WWP2 by Dvl2 is enhanced by a YxY element

NEDD4 family members are typically autoinhibited by an intramolecular interaction between the HECT domain and either the C2 domain (e.g. in Smurf2 and NEDD4) [27–29] or, in the case of Itch, sequences between the C2 and WW1 domains [25,30]. Ndfip proteins contain three PPxY elements in their cytoplasmic tail, and the disruption of the autoinhibitory conformation requires at least two of these to bind to WW domains [25,31]. Dvl2, however, contains only a single PPxY sequence, raising the question of whether this is sufficient to activate WWP2; residual binding of the Dvl2 PPxY mutant to WWP2 indicates that additional interactions must exist.

**Figure 3.**
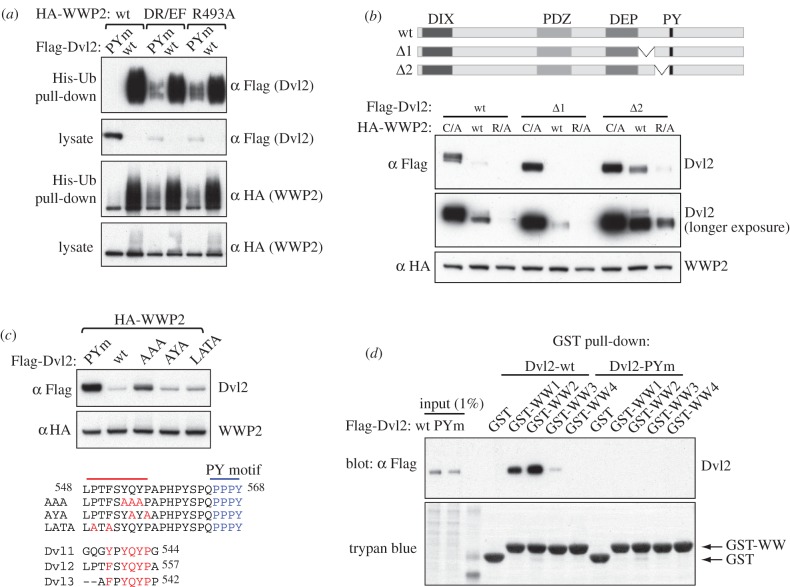
A conserved YxY element in Dvl2 contributes to its functional interaction with WWP2. (*a*) Western blot of His pull-downs from HEK293T cells co-transfected with His-Ub, Flag-Dvl2 and wt or mutant HA-WWP2, as indicated above panels; note the destabilization of PPxY-mutant Dvl2 by the WWP2 mutants bearing disinhibiting mutations in their HECT domains. (*b*,*c*) Western blots of HEK293T cell lysates after co-transfection with wt or mutant Flag-Dvl2 (as indicated above or underneath panels) and wt or mutant HA-WWP2 (R/A, R493A), revealing a conserved YxY motif 9 amino acids upstream of the PPxY element in Dvl2 (*c*) that contributes to its destabilization by disinhibited WWP2. (*d*) GST pull-down assays with individual GST-tagged WW domains from WWP2, from lysates of HEK293T transfected with wt or PPxY-mutant Dvl2, revealing a preference of its PPxY element for WW2 and WW1.

To verify these additional sites of interaction, we exploited a set of mutants of an invariant arginine in the WWP2 HECT domain. By analogy with the crystal structure of the related WWP1 HECT, which shows a compact and inactive conformation, this arginine is predicted to lie at the interface between the N lobe and C lobe (electronic supplementary material, figure S1). A natural mutation of WWP1 found in cancer cells maps to a similar interface location, though on an adjacent helix, and has been shown to cause constitutive activation of activity [32], perhaps by destabilizing this inactive conformation. As expected from this, we found that the arginine mutations in WWP2 partially relieved its autoinhibition, rendering it less dependent on Dvl2. Thus, unlike wild-type (wt) WWP2, these arginine mutants (DR/EF or R493A) are capable of ubiquitylating and destabilizing Dvl2-PYm (figure 3*a*), indicating at least one additional element outside PPxY that mediates recognition of Dvl2 by WWP2.

Because the activity of Ndfips involves multiple PPxY elements in close proximity, we first explored the sequences close to the Dvl2 PPxY motif. Deletion of residues C terminal to PPxY did not affect the WWP2-mediated destabilization of Dvl2 (electronic supplementary material, figure S2), nor did a small internal deletion on the N-terminal side of PPxY (Δ1); however, a deletion of the 28 amino acids immediately adjacent to PPxY (Δ2) reduced the destabilization of Dvl2 by wt WWP2, and to some extent also by R493A (figure 3*b*). Subsequent analysis identified a YxY motif as the functionally relevant element: a triple alanine substitution of YQY (AAA) recapitulated the effect of Δ2, blocking destabilization of Dvl2 to a significant extent, whereas other point mutations (including YAYA in which the glutamine of YQY and the downstream flanking proline are substituted) had very little effect (figure 3*c*). Notably, this YxY motif is conserved in Dvl1 and Dvl3 (figure 3*c*).

Given the proximity of YxY to the PPxY motif, which binds to WW domains, a likely site of interaction would be to the same or an adjacent WW domain. To test this, we expressed individual WW domains (tagged with GST) in bacteria and used these for pull-down assays of Dvl2 and Dvl2-PYm from lysates of transfected HEK293T cells. We found that wt Dvl2 bound WW2 and WW1 most strongly, and we could also detect a weak interaction with WW3, but not with WW4 (figure 3*d*). Thus, WW2 and WW1 appear to be the preferred domains for the PPxY motif of Dvl2. Binding of Dvl2-PYm was not detected under these conditions, indicating that if the YxY element binds WW domains at all, it does so very weakly on its own. It may therefore act mainly to enhance the PPxY–WW interaction, perhaps helping to position the PPxY element on a particular WW domain in the intact protein, rather than being an independent binding site.

### 2.4. The DEP domain of Dvl2 interacts with the C2 domain of WWP2

**Figure 4.**
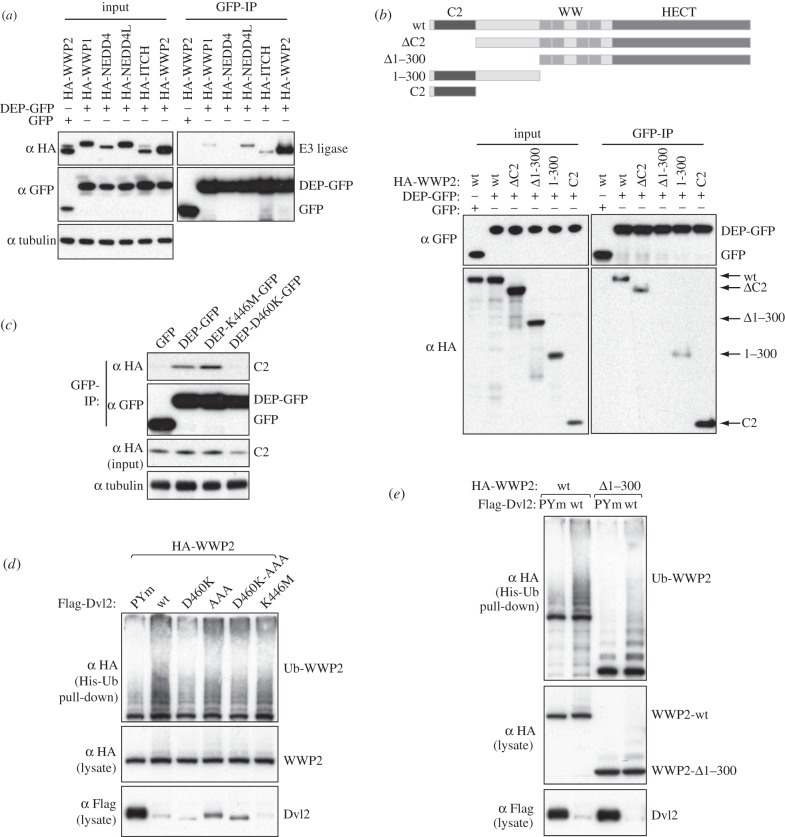
Functional interaction between the Dvl2 DEP domain and the WWP2 C2 domain. (*a*–*c*) Western blots of coIPs from HEK293T cells co-transfected with GFP-tagged DEP domain of Dvl2 and (*a*) different HA-tagged NEDD4 family ligases, (*b*) various truncations of HA-WWP2 and (*c*) HA-tagged C2 domain of WWP2, as indicated above panels. Dvl2-DEP exhibits a strong preference for WWP2 (*a*), and its binding to the WWP2 C2 domain (*b*) is blocked by a single point mutation (D460K) in a solvent-exposed surface residue within the second a helix of the DEP domain (*c*). (*d*) Western blot of His pull-downs from HEK293T cells co-transfected with His-Ub, wt or mutant Flag-Dvl2 and wt HA-WWP2, as indicated above panels. Note that D460K activates less well than wt, revealing a contribution of the Dvl2 DEP domain to the disinhibition of WWP2. (*e*) His pull-downs showing that wt and N-terminally truncated WWP2 are efficiently disinhibited by wt but not PPxY-mutant Dvl2.

Mutation of both the PPxY and YxY motifs was still not sufficient to prevent degradation of Dvl2 by the DR/EF-activated version of WWP2 (electronic supplementary material, figure S3), implying that there is at least one more way for WWP2 to interact with Dvl2. It has been reported that the association of Dvl1 with Itch partially depends on the DEP domain [13], so we tested interactions of this domain with WWP2. Indeed, a minimal DEP domain of Dvl2 tagged with green fluorescent protein (DEP–GFP) could coIP robustly with WWP2, and also weakly with Itch and NEDD4L (figure 4*a*). Mapping experiments revealed that the DEP domain binds to the C2 domain of WWP2, and apparently also weakly to sequences adjacent to it within the first 300 residues, but not to the WW or HECT domains (figure 4*b*). Furthermore, a point mutation within the DEP domain (D460K) blocked its interaction with the WWP2 C2 domain, whereas the well-known K446M point mutation (equivalent to the mutation in the *dsh*^1^ allele, which disrupts PCP signalling in flies and Frizzled-dependent membrane localization in mammalian cells) [33] did not affect it (figure 4*c*). This indicates a specific interaction between the Dvl2 DEP and the WWP2 C2 domains.

In several ligases, binding of the C2 domain to the HECT domain aids autoinhibition. Indeed, deletion of the C2 domain of WWP2 has been reported to activate this enzyme [28]. If binding of the Dvl2 DEP domain to the C2 domain competes with the HECT interaction, then it could lead to disinhibition. Consistent with this, we found that the D460K DEP mutant that blocks C2 binding was somewhat diminished in its ability to induce WWP2 autoubiquitylation, whereas the K446M mutant was not (figure 4*d*). In contrast, the AAA mutation (which removes the YxY motif) had little effect on WWP2 activation, even though it was a poorer substrate for degradation (figure 4*d*).

However, deletion of residues 1–300 of WWP2, which deletes both the C2 domain and adjacent sequences, yielded a construct that was still autoinhibited and could still be activated by Dvl2 in a PPxY-dependent manner, even though it could not bind to the DEP domain (figure 4*b*,*e*). The C2 domain cannot therefore be the only inhibitor of HECT activity. Interestingly, the related ligase WWP1 has also been reported to be inhibited both by its C2 domain and by the region encompassing the WW domains, though removal of any single WW domain did not relieve inhibition [32]. WWP2 behaved similarly—mutation of single WW domains had little effect, but mutation of multiple ones, notably WW2 and WW4, was sufficient to destabilize the full-length protein (electronic supplementary material, figure S4). Thus, sequences within or around the WW domains repress ligase activity, and binding of the PPxY motif to these is the major mechanism by which disinhibition occurs. The C2 domain appears to provide an additional or alternative mode of inhibition, which the DEP domain may relieve.

### 2.5. Disinhibition of WWP2 by Dvl2 depends on its polymerization

It remained puzzling that the single PPxY motif in Dvl2 could activate WWP2, given that previous studies with the Ndfip cytoplasmic domains had indicated a requirement for multiple PPxY elements for activation [25,31], and also that mutation of multiple WW domains is required to release inhibition. Recall, however, that overexpressed Dishevelled polymerizes to form cytoplasmic signalosomes, thereby attaining a local high concentration of ligand binding sites [7] including PPxY motifs. We thus conjectured that the local polymerization-dependent clustering of PPxY elements *in trans* could provide a similar function to their tandem *cis*-linkage in Ndfips. If so, the disinhibition of WWP2 by Dvl2 should depend on its ability to polymerize.

**Figure 5.**
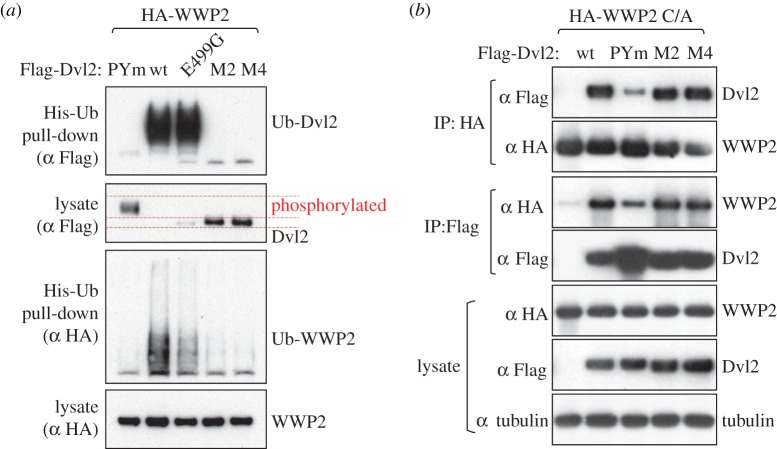
Disinhibition of WWP2 by Dvl2 depends on its polymerization. (*a*) Western blot of His pull-downs from HEK293T cells co-transfected with His-Ub, HA-WWP2 and wt or polymerization-deficient Flag-Dvl2 mutants (M2, M4), as indicated above panel; note the complete lack of activity of these mutants in disinhibiting WWP2. This is not due to lack of phosphorylation, because a phosphorylation-deficient DEP domain mutant (E499G) is nearly fully active. (*b*) Western blots of coIPs from HEK293ET cells co-transfected with catalytically inactive WWP2 and wt or mutant Flag-Dvl2, demonstrating that lack of polymerization does not affect the binding of Dvl2 to WWP2.

To test this, we co-expressed the polymerization-defective mutants M2 and M4 [9] with WWP2, and monitored their autoubiquitylation in His pull-down assays. Strikingly, neither of these mutants is ubiquitylated by WWP2, and both are therefore completely stable, like Dvl2-PYm (figure 5*a*). Furthermore, M2 and M4 are utterly incapable of disinhibiting WWP2 (figure 5*a*). This does not reflect their lack of binding to WWP2, because both mutants bind as efficiently as the wt to WWP2, in contrast to the PPxY mutant which binds less well (figure 5*b*). Thus, although unpolymerized Dvl2 can bind WWP2, polymerization is essential for its function to disinhibit the enzyme.

We noted that M2 and M4 migrated faster than wt Dvl2 on gels (figure 5*a*), a shift that has previously been shown to reflect lack of phosphorylation [12,34,35]. We considered the possibility that the key requirement is for polymerization-induced phosphorylation, rather than polymerization itself. Indeed, it has previously been noted that Itch- and Smurf2-dependent ubiquitylation and degradation of Dvl1 also correlate with its phosphorylation, and it was suggested that this might be a prerequisite [12,13]. However, a point mutant in the DEP domain (E499G) that was previously shown to lack phosphorylation [12] proved nearly as active as wt Dvl2 in disinhibiting WWP2 and was efficiently ubiquitylated and degraded (figure 5*a*). The D460K mutation described above also blocked phosphorylation, yet was efficiently degraded (figure 4*d* and electronic supplementary material, figure S5). We conclude that polymerization normally induces both phosphorylation of Dvl2 and disinhibition of WWP2, but phosphorylation is not essential for the disinhibition or for Dvl2 ubiquitylation.

Although polymerization is critical for both Dvl2 degradation and WWP2 activation, failure to polymerize does not explain the phenotypes of all the mutants we have described that show reduced activation: mutation of PPxY, or removal of the region encompassing the PPxY and YxY motifs, does not affect puncta formation (figure 1*b*) [26]. Similarly, the D460K mutant can form puncta, although the recruitment of WWP2 into these puncta is strongly reduced, as expected from the attenuated interaction of this mutant with the C2 domain (electronic supplementary material, figure S5). These mutations, therefore, define features required for polymerized (punctate) Dvl2 to bind and activate WWP2.

### 2.6. Autoubiquitylated WWP2 is stable

**Figure 6.**
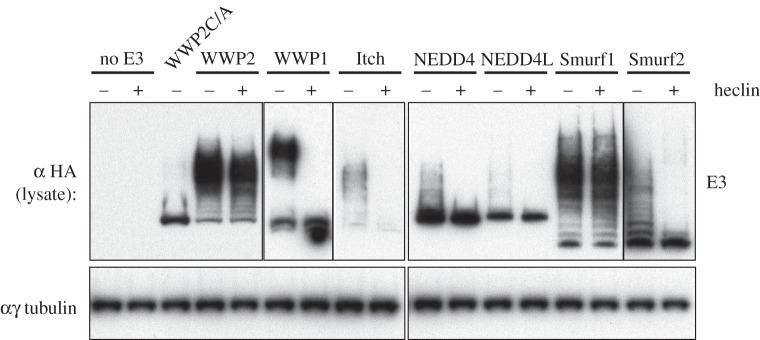
Stability of autoubiquitylated WWP2. Western blot of HEK293T cell lysates after transfection with HA-tagged NEDD4 family ligases (plus Ndfip, for maximal disinhibition of their E3 activity), with or without 100 µM heclin inhibitor for 2 h before lysis, revealing perdurance of autoubiquitylated WWP2 and Smurf1, in contrast to the other E3 ligases whose ubiquitylated forms are unstable under these conditions.

Activation of the autoubiquitylation of WWP2 might be expected to lead to its rapid degradation, thus limiting any consequences of ligase activation. Surprisingly, however, we observed no Dvl2-dependent reduction of WWP2 levels. To investigate this further, we followed the fate of the autoubiquitylated ligase after its activity was blocked with the compound heclin, which inhibits a broad range of HECT ligases including WWP2 [16], and thus prevents any new autoubiquitylation. For comparison, we tested several NEDD4 family ligases, all activated with Ndfip2. Figure 6 shows that 2 h after heclin addition, ubiquitylated forms of WWP1, Itch, NEDD4 and NEDD4L had disappeared, but ubiquitylated WWP2 remained largely stable, evidently avoiding the action of both deubiquitinases and the proteasome. Smurf1 also remained ubiquitylated, though the closely related Smurf2 did not. The stability of WWP2 contrasts with the fate of Dvl2, which as we have shown is degraded following modification by WWP2. We note, however, that in a similar heclin experiment Dvl2 appeared to be degraded more slowly when modified by WWP2 than by NEDD4 or Smurf2 [16]. In support of this, if the ratio of Dvl2 to co-expressed WWP2 is increased, then Dvl2 remains relatively stable despite being ubiquitylated by WWP2 (Michael Graeb, PhD thesis). Slow degradation could explain why ubiquitylated forms of Dvl2 are more prominent with WWP2 than with Itch or NEDD4, although all three ligases cause its degradation with comparable efficiency (figure 2*a*).

In general, differences in the stability of the ubiquitylated proteins, and in the precise accessibility of lysine residues in enzyme and substrate when they are bound together, may account for apparent anomalies in the behaviour of different enzymes and Dvl2 mutants. For example, Smurf1 appears to be well activated by Dvl2 but does not ubiquitylate it efficiently; conversely, Smurf2 and NEDD4 ubiquitylate Dvl2 and cause its destruction, but appear not to become autoubiquitylated in the process (figure 2*a*). The D460K Dvl2 mutation also shows reduced autoubiquitylation of WWP2, yet is efficiently degraded. Thus, although increased ubiquitylation is a sure sign of ligase activity, whether it occurs on the substrate, the enzyme or both is not always predictable.

The specific stability of ubiquitylated WWP2 and Smurf1 but not their substrates is striking. Why this should be is unclear, but it may reflect the configuration or length of the ubiquitin chains. Analysis of *in vitro* modified WWP2 (activated by Dvl2) by mass spectrometry revealed the presence of K63, K48 and some K11 linkages (see Methods), as others have found [22,36]. Interestingly, we were able to detect linkages to multiple lysine residues in the C2 domain (K17, 23, 28, 30, 54) and in WW4 (K453). Because these are domains implicated in autoinhibition, their ubiquitylation could potentially lead to a prolonged or more effective activation of WWP2.

### 2.7. The disinhibition of WWP2 by Dishevelled is conserved in flies

The strict dependence of the disinhibition of WWP2 on Dvl2 polymerization indicates that this process normally occurs in signalosomes, and in tissues that experience Wnt signalling. We thus wondered whether it is evolutionarily conserved, e.g. in *Drosophila* tissues where Dsh could be tested in physiological settings for its polymerization-dependent effects. *Drosophila* encodes four members of the NEDD4 family, one of each subgroup (NEDD4, Itch/WWP, Smurf and HECW/NEDL) [37], whereby the orthologue of WWP2 is Su(dx). Interestingly, genetic evidence implicates Su(dx) not in the attenuation of Wnt signalling but rather in the repression of Notch signalling [38], although there is partial redundancy with dNEDD4 and Smurf [39,40]. The most notable phenotype of these *Su(dx)^sp^* mutant flies is the vein gaps in their wings [38], which reflect hyperactive Notch signalling, but not increased levels of Dsh expression whose hallmark phenotype is supernumerary wing bristles [41]. This suggests that the primary physiological target of Su(dx) in developing fly wings is Notch rather than Dsh.

**Figure 7.**
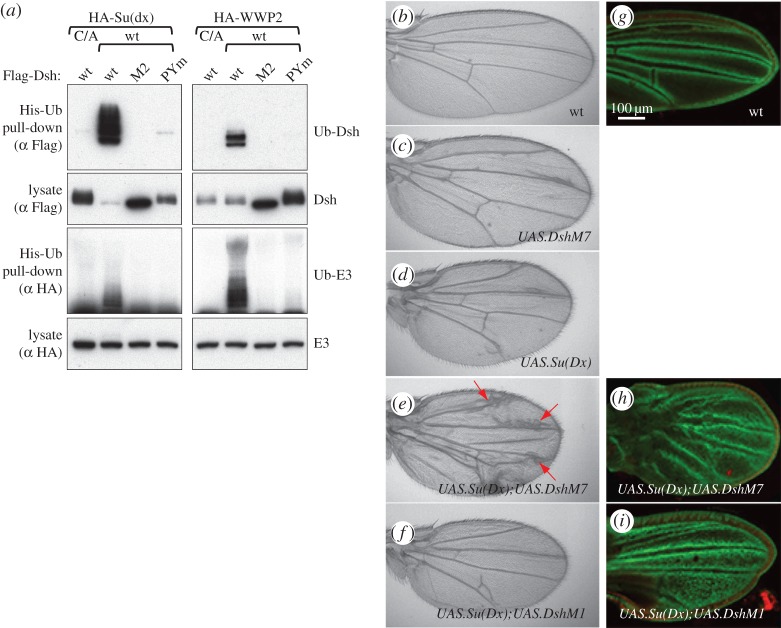
Disinhibition of Su(dx) by *Drosophila* Dishevelled. (*a*) Western blot of His pull-downs from HEK293T cells co-transfected with His-Ub, wt or catalytically inactive HA-WWP2 or HA-Su(dx), and wt or polymerization-deficient Flag-Dsh, as indicated above panels, revealing efficient disinhibition of both human and fly E3 ligase by *Drosophila* Dsh. (*b*–*f*) Fly wings after GAL4-mediated overexpression of Dsh and/or Su(dx) in the wing disc primordium during the third-larval instar (with MS1096.GAL4), as indicated in the panels; note the excess of vein material (arrows) after co-expression of polymerization-competent (DshM7) but not polymerization-deficient Dsh (DshM1), reflecting attenuated Notch signalling. Only mild vein defects are observed if Dsh or Su(dx) are overexpressed on their own. (*g*–*i*) Early pupal imaginal discs bearing the Notch reporter *E(spl)m*β*1.5-lacZ*, co-stained for lacZ (green) and DAPI (red) after co-expression of Dsh and Su(dx) as in (*b*), revealing strong defects in vein specification after co-expression of DshM7 but not DshM1. Scale bar, 100 µm.

To test whether Dsh can disinhibit Su(dx), like its human counterparts, we co-expressed Dsh, or its mutant versions Dsh-PYm and Dsh-M2, with Su(dx) or WWP2 in HEK293T cells. This confirmed that Dsh disinhibits Su(dx), thus inducing its own ubiquitylation and destabilization, but Dsh-M2 is completely inactive, and Dsh-PYm is also highly compromised (figure 7*a*). Therefore, the activity of Dishevelled in disinhibiting WWP2 in a polymerization-dependent way is conserved in flies. Dsh was also able to efficiently activate WWP2, but was itself modified with fewer ubiquitins and was not degraded as a result (as in the case of the catalytically inactive C/A mutant of WWP2, figure 7*a*). This may simply reflect the absence of suitably accessible lysine residues, but it emphasizes that activation does not necessarily require a good substrate (as noted above). The fact that both Dvl2 and Dsh are good substrates for the ligase of their own species suggests that they have evolved to be so.

To monitor the physiological consequences of the Dsh-induced disinhibition of Su(dx), we co-overexpressed these proteins in the wing imaginal disc. Expression of DshM7 alone (bearing the N80I point mutation in the DIX domain which does not affect polymerization, and barely attenuates Dsh function) [9,42] in late-stage wing discs causes merely mild wing defects, mostly occasional excess vein material (figure 7*c* compared with figure 7*b*), similarly to expression of Su(dx) on its own (figure 7*d*). This vein phenotype is massively exacerbated if the two proteins are co-overexpressed: we observe extensive vein broadening, especially near the wing margin zones where Dsh is activated by endogenous Wg signalling along the prospective margin (figure 7*e*). Vein broadening is consistent with a reduction in Notch signalling which normally refines broad pro-veins into the mature narrow wing veins [43], and is invariably detectable near the margin with weak loss-of-Notch conditions [44]. Importantly, this phenotypic exacerbation is not seen after co-expression of Su(dx) with DshM1 (a DIX domain point mutation that blocks polymerization and function of Dsh) [9,42]: these wings look essentially indistinguishable from the wings obtained after expression of Su(dx) on its own (figure 7*f*, compare with figure 7*d*). The same synergy between Su(dx) and DshM7, but not with the polymerization-defective DshM1, can already be seen in pupal wing discs if an *E(spl)m*β*1.5-lacZ* reporter [45] is monitored as a direct read-out of Notch signalling (figure 7*g–i*). Note that the epithelial cells of the pupal discs are completely undifferentiated at this early stage, which precedes the formation of mature veins by more than 2 days. These results strongly indicate that the disinhibition of Su(dx) by Dsh depends on its ability to polymerize, that this regulatory interaction between the two proteins is conserved in flies, and that a major target of activated Su(dx) is the Notch pathway.

### 2.8. Dvl2 stimulates WWP2-dependent ubiquitylation of Notch

Notch signalling results in intramembrane cleavage of the receptor (Notch) and release of its intracellular domain, which then moves to the nucleus and regulates transcription of specific genes. Intriguingly, a recent report identified WWP2 as a strong Notch3-interacting protein in a proteomics approach, and the authors showed that the intracellular domain of human Notch3 (NICD3) can be ubiquitylated in cells by co-expressed WWP2, although the activity was very weak [46]. This provides an obvious mechanism for the regulation of Notch3 by WWP2, and we wondered whether this activity could be enhanced by Dvl2.

**Figure 8.**
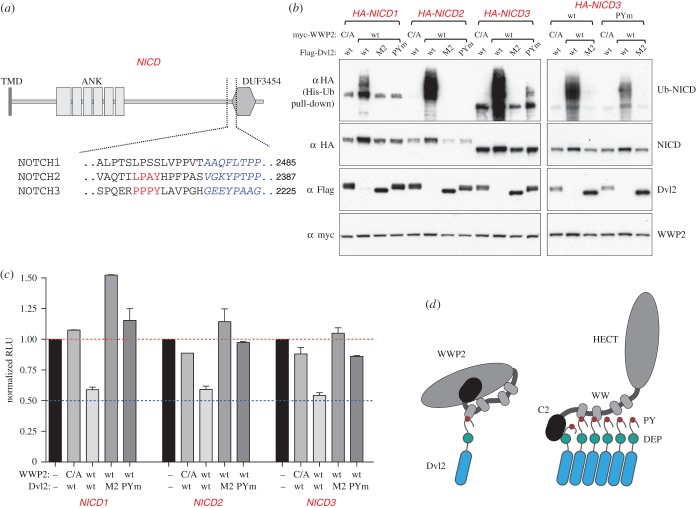
Ubiquitylation of Notch by Dvl2-activated WWP2 attenuates its activity. (*a*) Cartoon of the intracellular domain of Notch (NICD) and its domains, with PPxY elements and flanking sequences in human Notch2 and Notch3 underneath (no PPxY-like motifs are detectable in Notch1); TMD, transmembrane domain; ANK, ankyrin repeats; DUF3454, conserved domain of unknown function. (*b*) Western blot of His pull-downs from HEK293T cells co-transfected with His-Ub, wt HA-NICD (from Notch1, Notch2 and Notch3) or PPxY-mutant NICD (from Notch3), wt or catalytically inactive HA-WWP2 and wt, PPxY mutant (PYm) or polymerization-deficient (M2) Flag-Dvl2, as indicated above panels; note that efficient ubiquitylation of NICD conferred by WWP2 depends on its disinhibition by polymerization-competent Dvl2, on the PPxY element of Dvl2 and also, to some extent, on the PPxY element of NICD3. Note also that the low level of ubiquitylation of the PPxY-less NICD1 is comparable to the reduced level of NICD3 ubiquitylation in the absence of its PPxY, both of which are strictly dependent on polymerized Dvl2 (which may interact with NICD directly, see text). (*c*) Reporter assays in HEK293T cells co-transfected with a miminal Notch reporter (4xCBS-Luc), HA-NICD (from Notch1, Notch2 and Notch3), wt or catalytically inactive HA-WWP2 and wt or mutant Flag-Dvl2, revealing Dvl2-dependent reduction of the transcriptional activity of NICD. RLU, relative luciferase units (normalized to NICD-dependent activity in the absence of WWP2 and Dvl2); error bars, mean±s.d. (*d*) Disinhibition of WWP2 by polymerized Dishevelled: a model. *Left*, unpolymerized Dishevelled undergoes a single PPxY–WW interaction with WWP2, which is compatible with simultaneous autoinhibitory intramolecular C2–HECT and WW–HECT interactions. *Right*, DIX-mediated polymerization (triggered by Wnt stimulation, or high expression levels of Dishevelled) generates a high local concentration of Dishevelled, allowing WWP2 to undergo multiple simultaneous WW–PPxY and DEP–C2 interactions with adjacent Dishevelled molecules, which compete with the autoinhibitory intramolecular interactions of WWP2, thus exposing its HECT domain and allowing it to interact with E2 ligase and substrates.

To test this, we co-overexpressed the intracellular domains of Notch1, 2 and 3 with WWP2 and wt and mutant Dvl2 (M2 and PYm), and monitored their ubiquitylation by His pull-down assays. Notch3 contains a PPxY motif, which was shown to be required for its interaction with WWP2 [46]; Notch2 contains a related non-canonical LPAY sequence (figure 8*a*). Indeed, WWP2 ubiquitylated both NICD3 and NICD2—in each case, only if co-expressed with wt Dvl2 but not with the PYm or M2 mutants (figure 8*b*). Surprisingly, there was also some ubiquitylation of NICD1, even though this isoform lacks a PPxY motif. Mutating the PPxY motif of NICD3 reduced its ubiquitylation, but did not abolish it completely (figure 8*b*, right-hand side panel), implying an additional mode of recognition. This could reflect a second binding site on WWP2; however, evidence from *Drosophila* also suggests a direct interaction between Dvl and NICD [41,47,48], which would bring NICD into the proximity of Dishevelled-bound ligase. Thus, upon Wnt signalling, polymerized Dvl2 may act as a platform that not only recruits and activates the ubiquitin ligase, but also recruits a substrate, Notch. The implication is that Dvl2 can promote activity of WWP2 not only towards itself and Dvl2, but also towards other substrates, and the Notch intracellular domain has properties that make it a preferred target.

As with WWP2 itself, the ubiquitylation of the NICD domains by Dvl2-activated WWP2 did not reduce their levels; if anything, they were slightly increased. Nevertheless, the signalling activity of these domains was reduced (figure 8*c*), suggesting that ubiquitylation directly attenuates their nuclear function. This is consistent with the equivalent co-expression assays in fly wings (see above) where Dsh-activated Su(dx) causes a reduction in nuclear Notch signalling.

## 3. Discussion

Previous reports have shown that Dvl proteins associate with various members of the NEDD4 family through their PPxY motif, which leads to their ubiquitylation and subsequent proteasomal degradation [12–16]. By comparing these interactions side by side, we identified WWP2 as a preferred partner of Dvl2, binding not just to the PPxY motif but also through other interactions. For this reason, we have focused on WWP2, but other members of the NEDD4 family may share at least some of its properties, and indeed may have overlapping functions. We discovered that Dvl2 is not simply a substrate for WWP2 but can release the enzyme from its autoinhibition, and thus unlock its ubiquitin ligase activity towards itself, Dvl2 and an additional substrate, Notch. Strikingly, this activity of Dvl2 in disinhibiting WWP2 is totally dependent on its polymerization, which implies that this regulatory interaction will normally occur only when Dvl2 is within signalosomes.

HECT domains consist of two lobes that move relative to each other in the course of the ubiquitylation reaction, and autoinhibition involves intramolecular interactions that probably stabilize an inactive configuration of the HECT [27,28]. We have found with WWP2 that inhibition is mediated both by the C2 domain and by the region encompassing the WW domains, as also recently reported for WWP1 [32]. Dvl2 binds via its PPxY motif, and possibly the associated YxY sequence, to the WW domains and also, via its DEP domain, to the C2 domain, and is thus well equipped to disrupt the inhibitory state. Activation is most crucially dependent on two features of Dvl2: the PPxY motif and its polymerization. The effects of the latter are dramatic—though unpolymerized Dvl2, with its PPxY motif, can bind well to WWP2, it is completely unable to activate it. This phenomenon, together with the multiple interactions we have documented, suggests that the system has specifically evolved to switch on WWP2 activity in response to Dvl-dependent signalling. Furthermore, the key features are conserved between *Drosophila* and humans, suggesting an ancient mechanism. Our previous studies of the endosomal proteins Ndfip1 and 2 showed that multiple tandem PPxY motifs are required to activate HECT ligases, presumably by binding multiple WW domains and disrupting the inhibited conformation [25,31]. By analogy, we suggest that signalling-dependent polymerization of the Dishevelled proteins creates a local cluster of PPxY motifs that can bind simultaneously, or repeatedly, to multiple WW domains within a single WWP2 molecule, thus maintaining an open and active conformation (figure 8*d*).

What are the physiological consequences of the Dishevelled-mediated disinhibition of WWP2? Under the overexpression conditions that we and others have used, Dvl2 is efficiently ubiquitylated and degraded by the proteasome, and this could be an effective feedback mechanism to destroy polymerized Dvl2 and thus limit signalling. This negative feedback could be particularly important in post-mitotic cells, to limit unrestrained accumulation of Dishevelled over time which could trigger unwanted signalling through the canonical pathway. The effects may be subtle, however, or multiple ubiquitin ligases may be able to perform this role, as we have not observed any significant change in overall levels of Dvl in fly tissues when Su(dx) is mutated. Surprisingly, the induced autoubiquitylation of WWP2 (or its modification of Notch) does not result in degradation, suggesting that WWP2 generates ubiquitylations that are not good substrates for the proteasome. This questions the usual assumption that the self-inhibition of HECT ligases is required solely to prevent their degradation—for WWP2, it appears to have a regulatory role, and autoubiquitylation of inhibitory domains may, by preventing the re-formation of an inhibited state, prolong activity rather than reduce it.

There is strong evidence that one physiological target of the activated ligase is Notch. It was discovered a long time ago in flies that Dsh downregulates Notch signalling in developing wing tissue [41] and in other development contexts [47,48]. The genetics of the WWP2 homologue Su(dx) also implicates it in Notch downregulation [38]. Our study shows that the effects of Su(dx) in wing tissue are strongly enhanced by Dsh, in a polymerization-dependent manner. We can thus provide a plausible molecular explanation for the interaction between the Wnt and Notch pathways: Wnt signalling leads to Dsh polymerization, which activates Su(dx), which ubiquitylates Notch to downregulate its activity. This could occur either by induced endocytosis and degradation of intact Notch, thus reducing its availability at the plasma membrane for signalling, or by modification of the cleaved intracellular domain that results from signalling and abrogation of its transcriptional effects, as we have demonstrated in human cells. Such a process may be one of several mechanisms for cross-talk between these two signalling pathways—others have also been proposed, and may operate in addition [49]. However, our observation that mammalian Notch intracellular domains are good substrates for Dvl2-activated WWP2, via both PPxY-mediated and PPxY-independent mechanisms, together with previous observations that *Drosophila* Dsh can interact directly with Notch [41,47,48], suggests that Notch has evolved to be a prime target of Dishevelled-activated ligase and this property has been conserved during evolution. Whether there are also other regulatory proteins that are similarly ubiquitylated in response to Dishevelled signalosomes remains to be determined.

## 4. Material and methods

### 4.1. Plasmids

The following plasmids have been described before: human Flag-Dvl2, Flag-M2, Flag-M4, CMV-Renilla [26]; haemagglutinin (HA)-tagged NEDD4 family ligases (wt and catalytically inactive mutants), His-ubiquitin, Ndfip2 [16,25]; HA-Su(dx) [50]; Notch reporter 4xCBS-Luc (kindly provided by Sarah Bray). For bacterial expression in pGEX6P-2 (GE Healthcare, Piscataway, NJ), the following GST-WW domain constructs of human WWP2 were used: WW1 (301–333), WW2 (331–363), WW3 (406–437), WW4 (445–477). For mammalian expression, the following constructs were cloned into pcDNA3.1 (Invitrogen) in frame with a triple amino-terminal HA tag, an N-terminal EGFP plus triple HA-tag or a single N-terminal myc tag. Full-length WWP2 (1–870), C2-only (1–143), ΔC2 (144–870), WW domain point mutations in all combinations (WW1, W306G; WW2, W336G; WW3, W411G; WW4, W450G); mouse NICD1 (1747–2531), human NICD2 (1697–2471) and human NICD3 (1662–2321). Dvl2 constructs and mutants were made in the original Flag vector: Δ1 (508–535 deleted), Δ2 (536–563 deleted). DEP–GFP (414–506) was cloned into pEGFP-C2 (Clontech). Truncations and point mutations were generated by standard procedures and verified by sequencing. D460K was identified as a C2 non-binding mutant in a screen of eight point mutations in solvent-exposed conserved residues of the Dvl2 DEP domain (M. Gammons *et al*. 2015, manuscript in preparation). The partially disinhibited WWP2 mutant (DR492/493EF) was found accidentally.

### 4.2. Cell transfection and cell-based assays

HEK293T, HEK293ET and HeLa cells were cultured and transfected essentially as described [16,25,51]. Transient transfections of HEK293T cells were performed using polyethylenimine (PEI, linear, MW 25 000, Polysciences, Warrington, PA) or lipofectamine 2000 (Invitrogen) [51]. Typically, for 2 µg plasmid DNA, 5 µl of a 1 mg ml^−1^ solution of PEI was used, and cells were harvested 24 h after transfection. CoIPs and pull-downs were done essentially as described [16,25]. Briefly, for pull-downs or coIPs under native conditions, 1 × 10^6^ transfected cells were washed in phosphate-buffered saline (PBS) and subsequently lysed in lysis buffer (typically 20 mM Tris–HCl pH 7.4, 10% glycerol, 200 mM NaCl, 0.2% TritonX-100 supplemented with complete protease inhibitor cocktail and PhosSTOP phosphatase inhibitor cocktail, both from Roche) for 10 min on ice. Soluble proteins were recovered by centrifugation at 14 000*g* for 10 min at 4°C. For *α*-GFP IPs, 5 µl of GFP-Trap^®^ agarose beads (Chromotek, Planegg-Martinsried, Germany) was incubated with 500 µl lysate for 1 h at 4°C. Similarly, 20–40 µl of Flag-M2 affinity gel (Sigma) was used for *α*-Flag IPs. For pull-downs with GST-tagged recombinant proteins, the proteins were pre-bound to glutathione–sepharose (GE Healthcare) for 30 min before incubation with 500 µl lysate for 1 h at 4°C. The beads were then washed three times in lysis buffer before elution in 2× SDS–PAGE sample buffer and analysed by western blotting. His-ubiquitin pull-downs were done under denaturing conditions. Typically, 1 × 10^6^ cells were harvested in 0.8 ml 8 M urea, 100 mM Tris (pH 7.4), 2 mM *N*-ethylmaleimide, 10 mM iodoacetamide, sonicated and incubated with 10 µl His-tag Dynabeads (Life Technologies) at room temperature for several hours. The beads were washed three times in 8 M urea and eluted in sample buffer. Immunoblotting was done following standard procedures, and proteins were detected with antibodies from Sigma-Aldrich (HA, Flag M2, α or γ tubulin, myc) or Roche (GFP). For measuring NICD-dependent signalling activity, 0.5 × 10^6^ HEK293T cells were additionally transfected with the Notch reporter 4xCBS-Luc and CMV-renilla. Assay analysis was carried out 24 h after transfection using the Dual-Glo luciferase reporter assay (Promega) according to the manufacturer's protocol. Expression was normalized to Renilla luciferase. The data were expressed as mean ± s.d. For co-localization experiments, HeLa cells were transfected, fixed and stained as described [51].

### 4.3. Recombinant protein expression and purification

Expression and purification of recombinant proteins were done essentially as described [16]. Briefly, plasmids were transformed into BL21 (DE3) CodonPlus-competent *Escherichia coli* (Stratagene). Protein expression was induced with 0.5 mM isopropyl beta-d-thiogalactoside (IPTG) at 30°C for 3 h (individual WW domains) or 18°C overnight (full-length WWP2). Cell pellets were resuspended in PBS containing 1 mM TCEP and lysed by sonication. GST fusion proteins were affinity-purified from bacterial lysates by using glutathione–sepharose (GE Healthcare) according to standard manufacturer's protocols. After elution, recombinant proteins were immediately rebuffered in resuspension buffer containing 5–10% glycerol using PD-10 desalting columns (GE Healthcare), concentrated with Amicon Ultra centrifugation filter devices (Millipore) and stored as 5–10 mg ml^−1^ stocks at –80°C until required.

### 4.4. Fly assays

The following *Drosophila* strains were used: UAS.DshM1, UAS.DshM7 [42]; UAS.Su(dx) [52]; *E(spl)m*β*1.5-lacZ* [45]; MS1096.GAL4 (Flybase). Pupal wing discs were dissected, fixed and stained with α–β-galactosidase antibody (Promega) and DAPI (to control for the focal plane), as described [53]. Single confocal images were acquired at identical settings with a Zeiss confocal microscope. Fly wings were dissected and mounted by standard procedures, and images were taken under bright-field microscopy.

### 4.5. *In vitro* ubiquitylation and mass spectrometry

*In vitro* ubiquitylation experiments were done essentially as described [16]. Flag-Dvl2 was purified from transfected HEK293ET cells via anti-Flag IP followed by elution with Flag peptide. After *in vitro* ubiquitylation with UBE1, UbcH7, GST-WWP2 and ubiquitin for 3 h at 37°C, Dvl2 was re-purified by anti-Flag IP. After extensive washing, anti-Flag immunoprecipitates (Dvl2) and supernatant (containing WWP2) were separated by SDS–PAGE. The gel was cut into multiple slices for analysis by mass spectrometry. Briefly, the samples were reduced with 10 mM dithiothreitol, alkylated with 55 mM chloroacetamide, and digested overnight with 6 ng µl^−1^ trypsin (Promega, UK). The resulting peptides were extracted in 2% v/v formic acid, 2% v/v acetonitrile and analysed by LC–MS/MS with an ultimate U3000 HPLC (ThermoScientific Dionex, San Jose) after passaging over two successive C18 columns (ThermoScientific Dionex) and elution with a gradient of acetonitrile. The analytical column outlet was directly interfaced via a modified nano-flow electrospray ionization source, with a hybrid dual pressure linear ion trap mass spectrometer (Orbitrap Velos, ThermoScientific). MS spectra were collected over a *m*/*z* range of 300–2000, at 30 000 resolution. The tandem mass spectrometry (MS/MS) scans were collected using a threshold energy of 35 for collision-induced dissociation. LC–MS/MS data were then searched against a protein database (UniProt KB) using the Mascot search engine program (Matrix Science, UK).

## Supplementary Material

Supplementary figures

## References

[1] Clevers H 2006 Wnt/β-catenin signaling in development and disease. Cell 127, 469–480. (doi:10.1016/j.cell.2006.10.018) 17081971 10.1016/j.cell.2006.10.018

[2] Winston JT, Strack P, Beer-Romero P, Chu CY, Elledge SJ, Harper JW 1999 The SCFβ-TRCP-ubiquitin ligase complex associates specifically with phosphorylated destruction motifs in IκBβ and β-catenin and stimulates IκBβ ubiquitination *in vitro*. Genes Dev. 13, 270–283. (doi:10.1101/gad.13.3.270) 9990852 10.1101/gad.13.3.270 PMC316433

[3] Ha NC, Tonozuka T, Stamos JL, Choi HJ, Weis WI 2004 Mechanism of phosphorylation-dependent binding of APC to *β*-catenin and its role in *β*-catenin degradation. Mol. Cell 15, 511–521. (doi:10.1016/j.molcel.2004.08.010) 15327768 10.1016/j.molcel.2004.08.010

[4] Su Y, Fu C, Ishikawa S, Stella A, Kojima M, Shitoh K, Schreiber EM, Day BW, Liu B 2008 APC is essential for targeting phosphorylated *β*-catenin to the SCF*β*-TrCP ubiquitin ligase. Mol. Cell 32, 652–661. (doi:10.1016/j.molcel.2008.10.023) 19061640 10.1016/j.molcel.2008.10.023

[5] Mendoza-Topaz C, Mieszczanek J, Bienz M 2011 The Adenomatous polyposis coli tumour suppressor is essential for Axin complex assembly and function and opposes Axin's interaction with Dishevelled. Open Biol. 1, 110013 (doi:10.1098/rsob.110013) 22645652 10.1098/rsob.110013 PMC3352083

[6] MacDonald BT, Tamai K, He X 2009 Wnt/β-catenin signaling: components, mechanisms, and diseases. Dev. Cell 17, 9–26. (doi:10.1016/j.devcel.2009.06.016) 19619488 10.1016/j.devcel.2009.06.016 PMC2861485

[7] Bienz M 2014 Signalosome assembly by domains undergoing dynamic head-to-tail polymerization. Trends Biochem. Sci. 39, 487–495. (doi:10.1016/j.tibs.2014.08.006) 25239056 10.1016/j.tibs.2014.08.006

[8] Angers S, Moon RT 2009 Proximal events in Wnt signal transduction. Nat. Rev. Mol. Cell Biol. 10, 468–477. (doi:10.1038/nrn2674) 19536106 10.1038/nrm2717

[9] Schwarz-Romond T, Fiedler M, Shibata N, Butler PJ, Kikuchi A, Higuchi Y, Bienz M. 2007 The DIX domain of Dishevelled confers Wnt signaling by dynamic polymerization. Nat. Struct. Mol. Biol. 14, 484–492. (doi:10.1038/nsmb1247) 17529994 10.1038/nsmb1247

[10] Angers S, Thorpe CJ, Biechele TL, Goldenberg SJ, Zheng N, MacCoss MJ, Moon RT. 2006 The KLHL12-Cullin-3 ubiquitin ligase negatively regulates the Wnt-β-catenin pathway by targeting Dishevelled for degradation. Nat. Cell Biol. 8, 348–357. (doi:10.1038/ncb1381) 16547521 10.1038/ncb1381

[11] Ganner A et al. 2009 Regulation of ciliary polarity by the APC/C. Proc. Natl Acad. Sci. USA 106, 17 799–17 804. (doi:10.1073/pnas.0909465106) 10.1073/pnas.0909465106 PMC2764926 19805045

[12] Narimatsu M et al. 2009 Regulation of planar cell polarity by Smurf ubiquitin ligases. Cell 137, 295–307. (doi:10.1016/j.cell.2009.02.025) 19379695 10.1016/j.cell.2009.02.025

[13] Wei W, Li M, Wang J, Nie F, Li L 2012 The E3 ubiquitin ligase ITCH negatively regulates canonical Wnt signaling by targeting Dishevelled protein. Mol. Cell Biol. 32, 3903–3912. (doi:10.1128/MCB.00251-12) 22826439 10.1128/MCB.00251-12 PMC3457526

[14] Ding Y, Zhang Y, Xu C, Tao QH, Chen YG 2013 HECT domain-containing E3 ubiquitin ligase NEDD4L negatively regulates Wnt signaling by targeting Dishevelled for proteasomal degradation. J. Biol. Chem. 288, 8289–8298. (doi:10.1074/jbc.M112.433185) 23396981 10.1074/jbc.M112.433185 PMC3605647

[15] Nethe M et al. 2012 Rac1 acts in conjunction with Nedd4 and Dishevelled-1 to promote maturation of cell–cell contacts. J. Cell Sci. 125, 3430–3442. (doi:10.1242/jcs.100925) 22467858 10.1242/jcs.100925 PMC3516380

[16] Mund T, Lewis MJ, Maslen S, Pelham HR 2014 Peptide and small molecule inhibitors of HECT-type ubiquitin ligases. Proc. Natl Acad. Sci. USA 111, 16 736–16 741. (doi:10.1073/pnas.1412152111) 10.1073/pnas.1412152111 PMC4250122 25385595

[17] Rothbacher U, Laurent MN, Deardorff MA, Klein PS, Cho KW, Fraser SE 2000 Dishevelled phosphorylation, subcellular localization and multimerization regulate its role in early embryogenesis. EMBO J. 19, 1010–1022. (doi:10.1093/emboj/19.5.1010) 10698942 10.1093/emboj/19.5.1010 PMC305640

[18] Yanfeng WA, Berhane H, Mola M, Singh J, Jenny A, Mlodzik M 2011 Functional dissection of phosphorylation of Disheveled in *Drosophila*. Dev. Biol. 360, 132–142. (doi:10.1016/j.ydbio.2011.09.017) 21963539 10.1016/j.ydbio.2011.09.017 PMC3221411

[19] Gonzalez-Sancho JM, Greer YE, Abrahams CL, Takigawa Y, Baljinnyam B, Lee KH, Lee KS, Rubin JS, Brown AMC. 2013 Functional consequences of Wnt-induced Dishevelled 2 phosphorylation in canonical and noncanonical Wnt signaling. J. Biol. Chem. 288, 9428–9437. (doi:10.1074/jbc.M112.448480) 23396967 10.1074/jbc.M112.448480 PMC3611012

[20] de Groot RE et al. 2014 Huwe1-mediated ubiquitylation of Dishevelled defines a negative feedback loop in the Wnt signaling pathway. Sci. Signal. 7, ra26. (doi:10.1126/scisignal.2004985) 10.1126/scisignal.2004985 24643799

[21] Madrzak J, Fiedler M, Johnson CM, Ewan R, Knebel A, Bienz M, Chin JW. 2015 Ubiquitination of the Dishevelled DIX domain blocks its head-to-tail polymerization. Nat. Commun. 6, 6718 (doi:10.1038/ncomms7718) 25907794 10.1038/ncomms7718 PMC4423210

[22] Sheng Y et al. 2012 A human ubiquitin conjugating enzyme (E2)-HECT E3 ligase structure-function screen. Mol. Cell Proteomics 11, 329–341. (doi:10.1074/mcp.O111.013706) 22496338 10.1074/mcp.O111.013706 PMC3412965

[23] Jung H et al. 2013 Deubiquitination of Dishevelled by Usp14 is required for Wnt signaling. Oncogenesis 2, e64 (doi:10.1038/oncsis.2013.28) 23958854 10.1038/oncsis.2013.28 PMC3759127

[24] Tauriello DV, Haegebarth A, Kuper I, Edelmann MJ, Henraat M, Canninga-van Dijk MR, Kessler BM, Clevers H, Maurice MM. 2010 Loss of the tumor suppressor CYLD enhances Wnt/β-catenin signaling through K63-linked ubiquitination of Dvl. Mol. Cell 37, 607–619. (doi:10.1016/j.molcel.2010.01.035) 20227366 10.1016/j.molcel.2010.01.035

[25] Mund T, Pelham HR 2009 Control of the activity of WW-HECT domain E3 ubiquitin ligases by NDFIP proteins. EMBO Rep. 10, 501–507. (doi:10.1038/embor.2009.30) 19343052 10.1038/embor.2009.30 PMC2680872

[26] Schwarz-Romond T, Metcalfe C, Bienz M 2007 Dynamic recruitment of axin by Dishevelled protein assemblies. J. Cell Sci. 120, 2402–2412. (doi:10.1242/jcs.002956) 17606995 10.1242/jcs.002956

[27] Mari S, Ruetalo N, Maspero E, Stoffregen MC, Pasqualato S, Polo S, Wiesner S. 2014 Structural and functional framework for the autoinhibition of Nedd4-family ubiquitin ligases. Structure 22, 1639–1649. (doi:10.1016/j.str.2014.09.006) 25438670 10.1016/j.str.2014.09.006

[28] Wiesner S, Ogunjimi AA, Wang HR, Rotin D, Sicheri F, Wrana JL, Forman-Kay JD. 2007 Autoinhibition of the HECT-type ubiquitin ligase Smurf2 through its C2 domain. Cell 130, 651–662. (doi:10.1016/j.cell.2007.06.050) 17719543 10.1016/j.cell.2007.06.050

[29] Wang J, Peng Q, Lin Q, Childress C, Carey D, Yang W 2010 Calcium activates Nedd4 E3 ubiquitin ligases by releasing the C2 domain-mediated auto-inhibition. J. Biol. Chem. 285, 12279–12288. (doi:10.1074/jbc.M109.086405) 20172859 10.1074/jbc.M109.086405 PMC2852967

[30] Gallagher E, Gao M, Liu YC, Karin M 2006 Activation of the E3 ubiquitin ligase Itch through a phosphorylation-induced conformational change. Proc. Natl Acad. Sci. USA 103, 1717–1722. (doi:10.1073/pnas.0510664103) 16446428 10.1073/pnas.0510664103 PMC1413664

[31] Riling C, Kamadurai H, Kumar S, O‘Leary CE, Wu K-P, Manion EE, Ying M, Schulman BA, Oliver PM 2015 Itch WW domains inhibit its E3 ubiquitin ligase activity by blocking E2-E3 transthiolation. J. Biol. Chem. 290, 23 857–23 887. (doi:10.1074/jbc.M115.649269) 10.1074/jbc.M115.649269 PMC4583040 26245901

[32] Courivaud T, Ferrand N, Elkhattouti A, Kumar S, Levy L, Ferrigno O, Atfi A, Prunier C. 2015 Functional characterization of a WWP1/Tiul1 tumor-derived mutant reveals a paradigm of its constitutive activation in human cancer. J. Biol. Chem. 290, 21007–21018. (doi:10.1074/jbc.M115.642314) 26152726 10.1074/jbc.M115.642314 PMC4543659

[33] Yu A, Xing Y, Harrison SC, Kirchhausen T 2010 Structural analysis of the interaction between Dishevelled2 and clathrin AP-2 adaptor, a critical step in noncanonical Wnt signaling. Structure 18, 1311–1320. (doi:10.1016/j.str.2010.07.010) 20947020 10.1016/j.str.2010.07.010 PMC2992793

[34] Yanagawa S, van Leeuwen F, Wodarz A, Klingensmith J, Nusse R 1995 The Dishevelled protein is modified by wingless signaling in *Drosophila*. Genes Dev. 9, 1087–1097. (doi:10.1101/gad.9.9.1087) 7744250 10.1101/gad.9.9.1087

[35] Bernatik O, Sedova K, Schille C, Ganji RS, Cervenka I, Trantirek L, Schambony A, Zdrahal Z, Bryja V. 2014 Functional analysis of Dishevelled-3 phosphorylation identifies distinct mechanisms driven by casein kinase 1 and Frizzled5. J. Biol. Chem. 289, 23 520–23 533. (doi:10.1074/jbc.M114.590638) 10.1074/jbc.M114.590638 PMC4156093 24993822

[36] Hong JH, Ng D, Srikumar T, Raught B 2015 The use of ubiquitin lysine mutants to characterize E2–E3 linkage specificity: mass spectrometry offers a cautionary ‘tail’. Proteomics 15, 2910–2915. (doi:10.1002/pmic.201500058) 26036340 10.1002/pmic.201500058

[37] Marin I 2010 Animal HECT ubiquitin ligases: evolution and functional implications. BMC Evol. Biol. 10, 56 (doi:10.1186/1471-2148-10-56) 20175895 10.1186/1471-2148-10-56 PMC2837046

[38] Mazaleyrat SL, Fostier M, Wilkin MB, Aslam H, Evans DA, Cornell M, Baron M. 2003 Down-regulation of Notch target gene expression by Suppressor of deltex. Dev. Biol. 255, 363–372. (doi:10.1016/S0012-1606(02)00086-6) 12648496 10.1016/s0012-1606(02)00086-6

[39] Wilkin MB et al. 2004 Regulation of Notch endosomal sorting and signaling by *Drosophila* Nedd4 family proteins. Curr. Biol. 14, 2237–2244. (doi:10.1016/j.cub.2004.11.030) 15620650 10.1016/j.cub.2004.11.030

[40] Sakata T, Sakaguchi H, Tsuda L, Higashitani A, Aigaki T, Matsuno K, Hayashi S. 2004 *Drosophila* Nedd4 regulates endocytosis of Notch and suppresses its ligand-independent activation. Curr. Biol. 14, 2228–2236. (doi:10.1016/j.cub.2004.12.028) 15620649 10.1016/j.cub.2004.12.028

[41] Axelrod JD, Matsuno K, Artavanis-Tsakonas S, Perrimon N 1996 Interaction between Wingless and Notch signaling pathways mediated by Dishevelled. Science 271, 1826–1832. (doi:10.1126/science.271.5257.1826) 8596950 10.1126/science.271.5257.1826

[42] Penton A, Wodarz A, Nusse R 2002 A mutational analysis of Dishevelled in *Drosophila* defines novel domains in the Dishevelled protein as well as novel suppressing alleles of axin. Genetics 161, 747–762. 12072470 10.1093/genetics/161.2.747 PMC1462152

[43] Blair SS 2007 Wing vein patterning in *Drosophila* and the analysis of intercellular signaling. Annu. Rev. Cell Dev. Biol. 23, 293–319. (doi:10.1146/annurev.cellbio.23.090506.123606) 17506700 10.1146/annurev.cellbio.23.090506.123606

[44] Parody TR, Muskavitch MA 1993 The pleiotropic function of Delta during postembryonic development of *Drosophila melanogaster*. Genetics 135, 527–539. 8244012 10.1093/genetics/135.2.527 PMC1205653

[45] Cooper MT, Tyler DM, Furriols M, Chalkiadaki A, Delidakis C, Bray S 2000 Spatially restricted factors cooperate with Notch in the regulation of *Enhancer of split* genes. Dev. Biol. 221, 390–403. (doi:10.1006/dbio.2000.9691) 10790334 10.1006/dbio.2000.9691

[46] Jung JG, Stoeck A, Guan B, Wu RC, Zhu H, Blackshaw S, Shih I-M, Wang T-L. 2014 Notch3 interactome analysis identified WWP2 as a negative regulator of Notch3 signaling in ovarian cancer. PLoS Genet. 10, e1004751 (doi:10.1371/journal.pgen.1004751) 25356737 10.1371/journal.pgen.1004751 PMC4214668

[47] Capilla A, Johnson R, Daniels M, Benavente M, Bray SJ, Galindo MI 2012 Planar cell polarity controls directional Notch signaling in the *Drosophila* leg. Development 139, 2584–2593. (doi:10.1242/dev.077446) 22736244 10.1242/dev.077446 PMC3383230

[48] Ramain P, Khechumian K, Seugnet L, Arbogast N, Ackermann C, Heitzler P 2001 Novel Notch alleles reveal a Deltex-dependent pathway repressing neural fate. Curr. Biol. 11, 1729–1738. (doi:10.1016/S0960-9822(01)00562-0) 11719214 10.1016/s0960-9822(01)00562-0

[49] Collu GM, Hidalgo-Sastre A, Brennan K 2014 Wnt–Notch signalling crosstalk in development and disease. Cell. Mol. Life Sci. 71, 3553–3567. (doi:10.1007/s00018-014-1644-x) 24942883 10.1007/s00018-014-1644-x PMC11113451

[50] Djiane A, Shimizu H, Wilkin M, Mazleyrat S, Jennings MD, Avis J, Bray S, Baron M. 2011 Su(dx) E3 ubiquitin ligase-dependent and -independent functions of Polychaetoid, the *Drosophila* ZO-1 homologue. J. Cell Biol. 192, 189–200. (doi:10.1083/jcb.201007023) 21200027 10.1083/jcb.201007023 PMC3019562

[51] Metcalfe C, Mendoza-Topaz C, Mieszczanek J, Bienz M 2010 Stability elements in the LRP6 cytoplasmic tail confer efficient signalling upon DIX-dependent polymerization. J. Cell Sci. 123, 1588–1599. (doi:10.1242/jcs.067546) 20388731 10.1242/jcs.067546 PMC2858023

[52] Cornell M et al. 1999 The *Drosophila melanogaster* *Suppressor of deltex* gene, a regulator of the Notch receptor signaling pathway, is an E3 class ubiquitin ligase. Genetics 152, 567–576. 10353900 10.1093/genetics/152.2.567 PMC1460625

[53] Pi H, Huang YC, Chen IC, Lin CD, Yeh HF, Pai LM 2011 Identification of 11-amino acid peptides that disrupt Notch-mediated processes in *Drosophila*. J. Biomed. Sci. 18, 42 (doi:10.1186/1423-0127-18-42) 21682860 10.1186/1423-0127-18-42 PMC3136413

