# Supplementary material for: Disinhibition of the HECT E3 ubiquitin ligase WWP2 by polymerized Dishevelled
Source: Open Biol. 2015 Dec 23;5(12):150185. doi: 10.1098/rsob.150185 (PMC4703060; doi:10.1098/rsob.150185)
Supplement: Supplementary figures [file rsob150185supp1.pdf]

Supplementary Figures

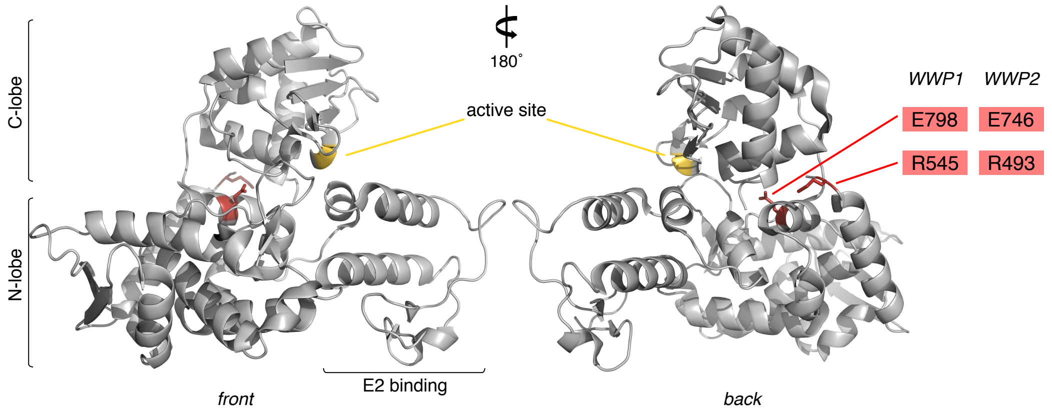

Fig. S1. The crystal structure of the WWP1 HECT domain (1ND7). During catalysis, the C lobe oscillates between a position where it can receive Ub from the E2 to one where it can transfer Ub to substrate. The WWP1 structure is compact, with the active site Cys residue relatively buried and not in a position to receive Ub from E2, which binds to the front of the indicated domain. Stabilisation of such a structure is a possible means of auto-inhibition. The activating mutation found in WWP1 in cancer cells (E798V) removes a charge interaction at the interface between the C and N lobes. The Arg residue mutated in WWP2 (R493A, corresponding to R545 in WWP1) would be at the position indicated, which would allow interaction with acidic residues on the C lobe. We suggest that loosening this interaction makes release of the C lobe from this conformation easier and thus weakens auto-inhibition.

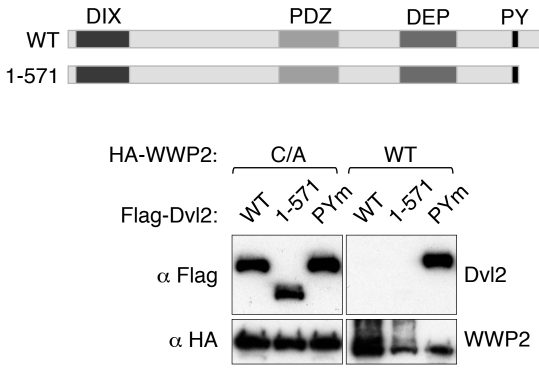

Fig. S2. Deletion of Dvl2 sequences C terminal to the PY motif does not prevent degradation of Dvl2 or activation of WWP2. Western blots of lysates from cells expressing the indicated proteins are shown. Panels are different lanes from the same exposure of the same gel.

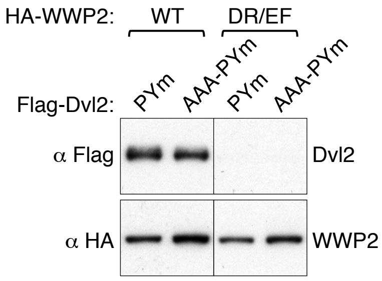

Fig. S3. Mutation of both the PPxY motif and the nearby YxY sequence (AAA mutation) does not prevent degradation of Dvl2 by an activated version of WWP2 (DR/EF). Western blots of co-transfected cell lysates are shown (left and right panels are lanes from the same exposure of a single gel).

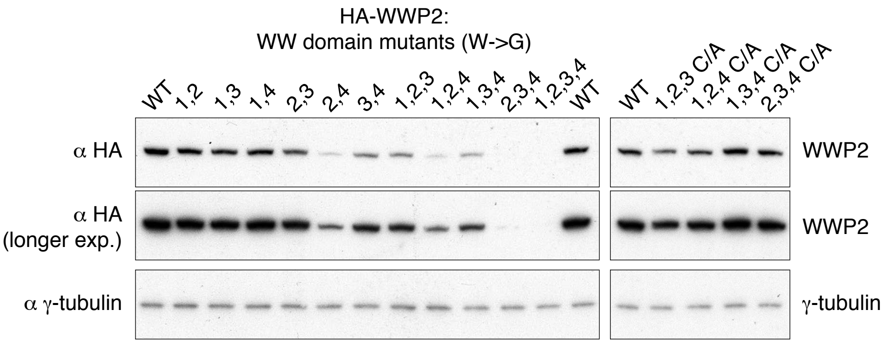

Fig. S4. Multiple WW domains contribute to auto-inhibition of WWP2. Point mutations were introduced into one or more WW domains by changing the first conserved W to G. Constitutively active mutants, in contract to wt protein activated by Dvl2, undergo degradation and thus activity can be inferred from the level of WWP2 detected. That loss of protein is due to WWP2 activity is demonstrated by the stability of corresponding WW mutant forms of the catalytically inactive C/A form (right hand lanes). Most double mutants remain stable but there is marked instability of all those in which both WW2 and WW4 are mutated.

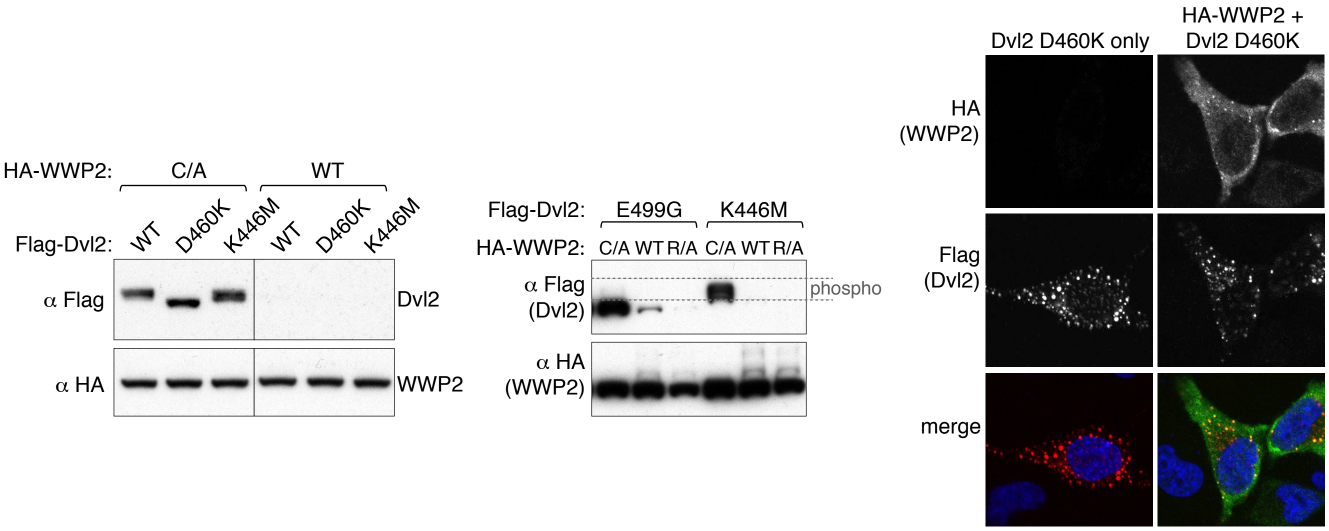

Fig. S5. Dvl2 DEP domain mutants D460K and E499G show reduced phosphorylation, but K446M does not. Increased mobility of E499G has previously been shown to be due to loss of phosphorylation, and these blots show that undegraded D460K and E499G (when expressed with catalytically inactive C/A WWP2) show this property, yet all three mutants are efficiently degraded by wt or activated (R/A) WWP2. Panels at right show that D460K still forms puncta when expressed in HeLa cells, but recruits WWP2 poorly (compare wt in Figure 1B).
